# Instrumental Occlusal Analysis in Migraine Patients: A Quantitative Cross Sectional Study

**DOI:** 10.1002/cre2.938

**Published:** 2024-07-23

**Authors:** Zokaris Nikolaos, Greven Marcus, Naoumis Dimitrios, Tzakis Michail, Mitsikostas Dimos Dimitrios, Psarras Vasileios

**Affiliations:** ^1^ 251 Hellenic Air Force and VA Hospital, Department of Prosthodontics Athens Greece; ^2^ Medical University of Vienna, University Clinic of Dentistry Vienna Austria; ^3^ Private Practice, Neurologist Athens Greece; ^4^ School of Dentistry, Department of Orofacial Pain National and Kapodistrian University of Athens Athens Greece; ^5^ School of Medicine, First Department of Neurology, Aeginition Hospital National and Kapodistrian University of Athens Athens Greece

**Keywords:** axiography, condylar displacement, instrumental functional analysis, instrumental occlusal analysis, jaw tracking, migraine, occlusion

## Abstract

**Objectives:**

This study aimed to investigate possible differences of functional occlusal variables between a group of migraine patients (MG) and a control group (CG).

**Materials and Methods:**

Each group included 50 individuals. Instrumental functional analysis and digital occlusal analysis were performed. Variables examined were condylar displacement from a reference position to maximum intercuspation; angular difference between the steepness of the articular eminence and the contra‐lateral canine guidance; and angular difference between the steepness of the articular eminence and the ipsilateral central incisor guidance and occlusal plane inclination. Self‐reported grinding and occlusal index were also investigated.

**Results:**

There were statistically significant differences in the extent of retral condylar displacement assessed both clinically [MG: 0.49 mm (SD 0.67 mm), CG: 0.29 mm (SD 0.27 mm), *p* = 0.012] and digital‐mechanically [MG: 1.53 mm (SD 0.95 mm), CG: 0.9 mm (SD 0.66 mm), *p* = 0.001], the angular difference between the steepness of the articular eminence and the contra‐lateral canine guidance [MG: 13.11° (SD 8.33°), CG: 9.47° (SD 7.08°), *p* = 0.021 and MG: 12.94° (SD 8.71°), CG: 9.44° (SD 8.70°), *p* = 0.017], and the occlusal plane inclination [MG: 11.16° (SD 4.66°), CG: 9.09° (SD 4.37°), *p* = 0.024]. Self‐reported grinding (MG: 39/50, CG: 12/50, *p* < 0.001) and occlusal index [MG: 1.92 (SD 0.46), CG: 0.21 (SD 0.66), *p* < 0.001] were also significantly higher in migraineurs.

**Conclusions:**

Articular and occlusal structures could play a role in migraine and thus should be considered in an interdisciplinary approach.

## 1 Introduction

Headaches are a common health issue. According to the World Health Organization, two to three‐quarters of adults aged 18–65 years have had headaches in the previous year (The World Health Organization 2011). Migraine affects around 15% of adults (Stovner and Andree 2010) and is among the most disabling diseases globally (Vos et al. 2016). Migraine is considered a neurovascular reaction to external and/or internal stimuli involving the trigeminovascular system. When activated, this system is thought to account for the pain and associated features of migraine (Sales Pinto et al. 2013). Numerous publications report a higher incidence of migraine in temporomandibular disorders (TMDs), cohorts and vice versa (Goncalves et al. 2013; Peşkersoy et al. 2016; Costa, D'Abreu, and Cendes 2008; Ciancaglini and Radaelli 2001; Glaros, Urban, and Locke 2007; Cooper and Kleinberg 2007; Ballegaard et al. 2008; Yoon et al. 2010; Franco et al. 2010; Gonçalves et al. 2010; Bevilaqua‐Grossi et al. 2010; Evans, Bassiur, and Schwartz 2011; Graff‐Radford and Abbott 2016; Ashraf et al. 2019), and an association between bruxism and migraine (Peşkersoy et al. 2016; Manfredini et al. 2013; Didier et al. 2014; Haggiag and Speciali 2020; Zaproudina, Lipponen, et al. 2018; Zaproudina, Rissanen, et al. 2018). In this threefold association, the role of occlusion cannot be overlooked. To our knowledge, only a few studies exist that have objectively investigated the importance of occlusal factors in pure migraine adult cohorts (Takeuchi et al. 2016; Kato et al. 2016; Takeuchi et al. 2013; Steele et al. 1991).

The importance of occlusion as the main causative factor for TMDs and/or orofacial pain has diminished over the last two decades. Occlusion is a sensitive neurologic feedback system that guides mandibular movements and a decisive factor for maintaining the condylar position within the physiological range of the limited functional joint space (Greven, Landry, and Carmignani 2016; Christiansen 2010). Occlusal conditions contributing to the violation of the functional joint space could raise brain activation of the prefrontal cortex and the amygdala, which are connected with emotional and painful neural processing (Otsuka et al. 2011; Greven et al. 2011). A dysfunctional interaction of the TMJ and the dental relationship can initiate a series of events caused by the sensory signals transmitted by the trigeminal nervous system into the central nervous system (CNS) (Demerjian, Barkhordarian, and Chiappelli 2018).

Proper equipment and methodology are paramount in the study of occlusion. Various methodological flaws and the lack of proper instrumentation are among the reasons why the occlusal hypothesis is still being researched (Cordray 2017). The digital era provides the opportunity for a different insight into the study of occlusion. From a simple three‐dimensional (3D) scan of the dental arch to the dynamic localization of the mandibular hinge axis and the kinematic analysis of the mandible in the condylar level, contemporary instrumentation and software offer new, and possibly more accurate and efficient, approaches for studying old hypotheses.

The present study aimed to investigate and compare functional occlusal variables, using up‐to‐date digital instrumentation and software, in two matched by age and gender groups; the first group comprised of diagnosed migraine patients and the second group comprised of individuals without any headache involvement. Functional occlusal variables under investigation were (a) condylar displacement (CD) from a reference position (RP) to maximum intercuspation (ICP), both clinical and mechanical, (b) the angular difference between the steepness of the articular eminence and the contra‐lateral canine guidance, (c) the angular difference between the steepness of the articular eminence and the ipsilateral central incisor guidance, and (d) occlusal plane inclination (OPI). Two subjective parameters were also investigated: self‐reported grinding and clenching and occlusal index (OI) (Gsellmann et al. 1998). Thus, the null hypothesis formulated is that *Functional Occlusal variables do not differ between migraineurs and non‐headache sufferers.*

## 2 Materials and Methods

### 2.1 Sample Formation

For the migraine group (MG), consecutive patients of the outpatient headache clinic at Hellenic Air Force General and VA Hospital (251GNA), diagnosed with migraine disorders by a neurologist according to the ICHD‐3 criteria were requested to enroll in the study. Diagnoses that allowed enrollment were chronic migraine, migraine with and without aura, and probable migraine. Exclusion criteria for the MG were as follows:

- Use of complete or partial removable dentures.
- Active orthodontic treatment.
- Age < 18 years.
- Inability to understand basic English language.

For the Control group (CG), individuals attending the Department of Operative Dentistry at 251GNA for regular dental services and individuals from the hospital personnel were requested to enroll in the study.

The Migraine Disability Assessment (MIDAS) questionnaire (Stewart et al. 2001) was used for screening the CG. Thus, exclusion criteria for the control group were as follows:

- MIDAS score > 1.
- Missing occlusal contacts up to first molars.
- Use of complete or partial removable dentures.
- Active orthodontic treatment.
- Age < 18 years.
- Inability to understand basic English language.

The study was approved by the Scientific and Ethics Committee of 251GNA on 02/2020 (Φ.076/ΑΔ1348/Σ.48310.02.20/251ΓΝΑ). The last patient was enrolled on 10/2022.

All individuals received detailed information for the ongoing study and those who agreed to enroll voluntarily were referred to the Prosthodontic Department, where all parts of the study were carried out.

In order to calculate the minimum sample size, the power of the study was set at 80% and the confidence interval was set at 95%. Literature research was conducted in order to approximately define the incidence of each variable in both migraine and non‐headache sufferers. The online software https://clincalc.com/stats/samplesize.aspx was used to calculate the sample size. Depending on the variable, the sample size varied from 17 to 44 individuals. Accordingly, 50 individuals per group were selected as the appropriate sample size.

### 2.2 Research Workflow

On the first visit, all participants were informed in detail about the scope of the study, and a written consent was obtained. The MIDAS questionnaire was completed. Maxillary and mandibular dental arches were scanned using the TRIOS 3 intraoral scanner (3Shape, Copenhagen, Denmark) and casts were printed using Asiga Max UV 385 nm 3d printer (Asiga, Sydney, Australia). The mandibular cast was printed twice.

On the second visit, all participants underwent Instrumental Functional Analysis. Computerized axiography was performed with Cadiax 4 and the corresponding software Gamma Document Browser (Gamma Dental, Klosterneuburg, Austria). The dental and medical history provided by the software was completed. The computerized axiography (also referred to as condylography, jaw tracking, or instrumental motion analysis of the mandible) is a simple, noninvasive, and accurate method to observe and record the movement of the condylar hinge axis in the 3D coordinate system.

Reference position (RP) and intercuspal position (ICP) registrations were conducted with Variotime Bite (Heraus Kulzer, Hanau, Germany). The RP according to Slavicek is defined as the position in which “the mandible is in physiologic retral border position. All structures of the joint are unloaded, that is, the ligaments are not in tension in any direction. There is only minimum muscle activity and no pressure on cartilaginous structures” (Piehslinger et al. 1993). For the RP registration, an anterior deprogrammer (AD) was fabricated by Pattern Resin (GC America Inc, Alsip, IL, USA) with a flat occlusal surface and only one contact point as close as possible to the midline, leaving minimal space between the dental arches. The AD was worn for at least 7–10 min before the RP registration, ensuring that no tooth contact occurred. Just before the registration, with the AD in place and no tooth contact possible, the patient was asked to move the mandible forward, backward, left, and right in order to lubricate the joint. No attempt was made to manipulate the subject's mandible into position; chin control (no manipulation to the patient/patient active technique) with no pressure ensured a smooth pressure‐less closure.

Printed casts were mounted at the Reference SL articulator (Gamma Dental, Klosterneuberg, Austria). The maxillary cast was mounted directly after jaw tracking to the actual hinge axis, while one mandibular cast was mounted in RP and a second in ICP. The reference plane was thus the axis–orbital plane. The articulator with the mounted casts was scanned using the Zirkonzahn S900 Arti scanner (Zirkonzahn, Gais, Italy), so that data could be imported to Cadias 3D software to analyze the occlusal features. For each subject, two laboratory scans were required: one in RP and one in ICP; thus, three scanned models were finally imported: maxillary model, mandibular model in RP, and mandibular model in ICP.

### 2.3 Occlusal Variables

**Figure 1:**
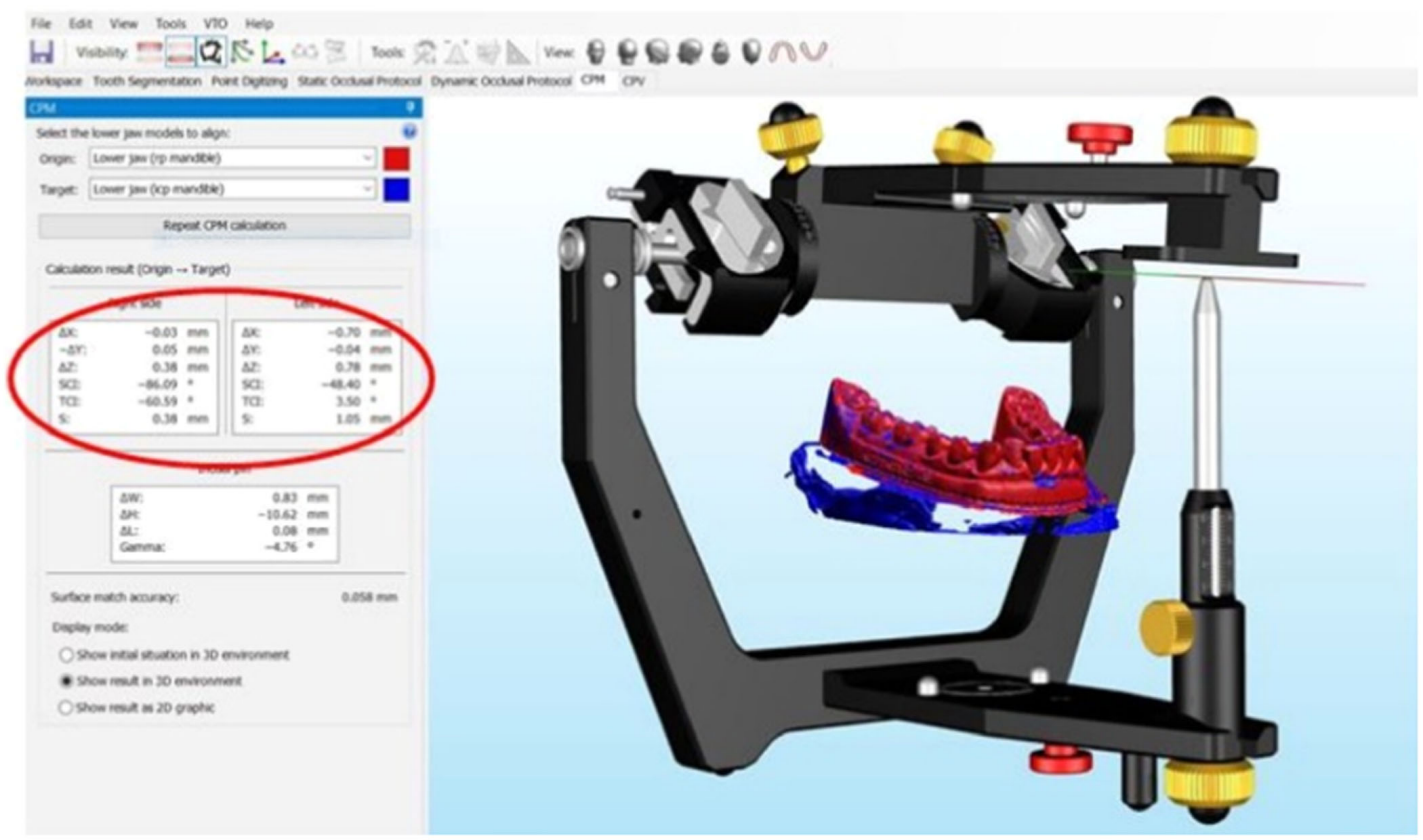
Mechanical condylar displacement evaluated in Cadias 3D software. In order to perform digital CPM, two lab scans are essential: one with the maxillary and the mandibular model in ICP and one with the maxillary model and the mandibular model in RP. The mandibular models are then aligned and condylar displacement from RP to ICP is calculated. The blue model represents the mandible in ICP. The red model represents the mandible in RP. The amount of displacement is shown to the left. ICP, maximum intercuspal position; RP, reference position.

**Figure 2:**
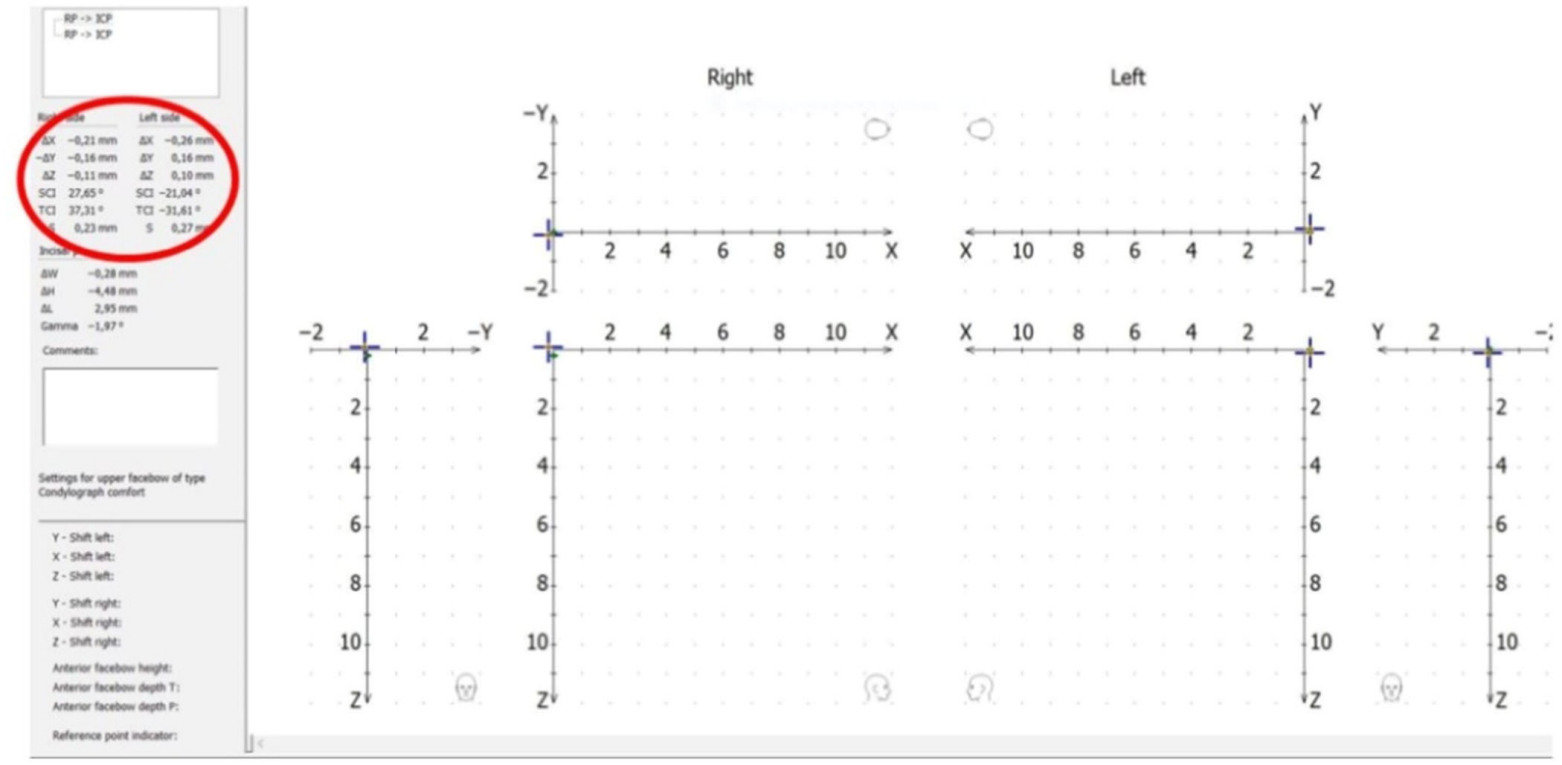
Clinical condylar displacement evaluated in jaw tracking. The patient is asked to close from RP to ICP. RP was achieved with an Anterior Deprogrammer in place for 7–10 min and unforced chin control. RP was the starting position. Immediately at the beginning of the recording, the Anterior Deprogrammer was removed and the patient was asked to close in ICP. The amount of displacement is shown directly to the left by the end of the recording. ICP, maximum intercuspal position; RP, reference position.

**Figure 3:**
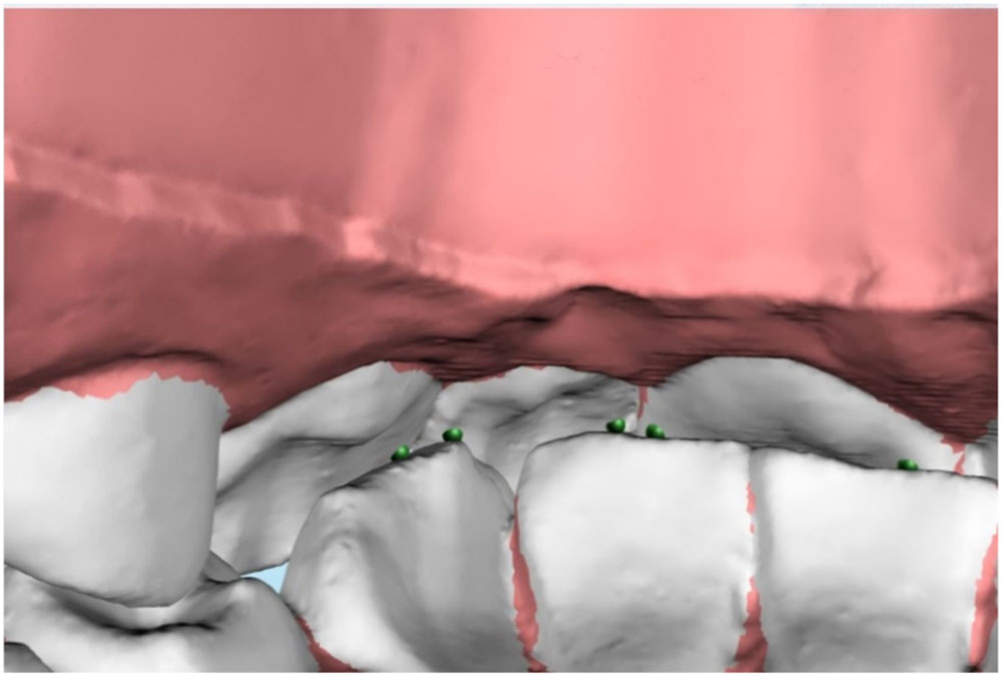
Example of F1 points corresponding to passive centric. The passive centric arch consists of the marginal crests of the anterior teeth, the canines, and premolars, along with the marginal crests and grooves of the molars of the maxilla. F1 represents the contact point of the antagonist and in anterior teeth, it is ideally located in the marginal crests.

**Figure 4:**
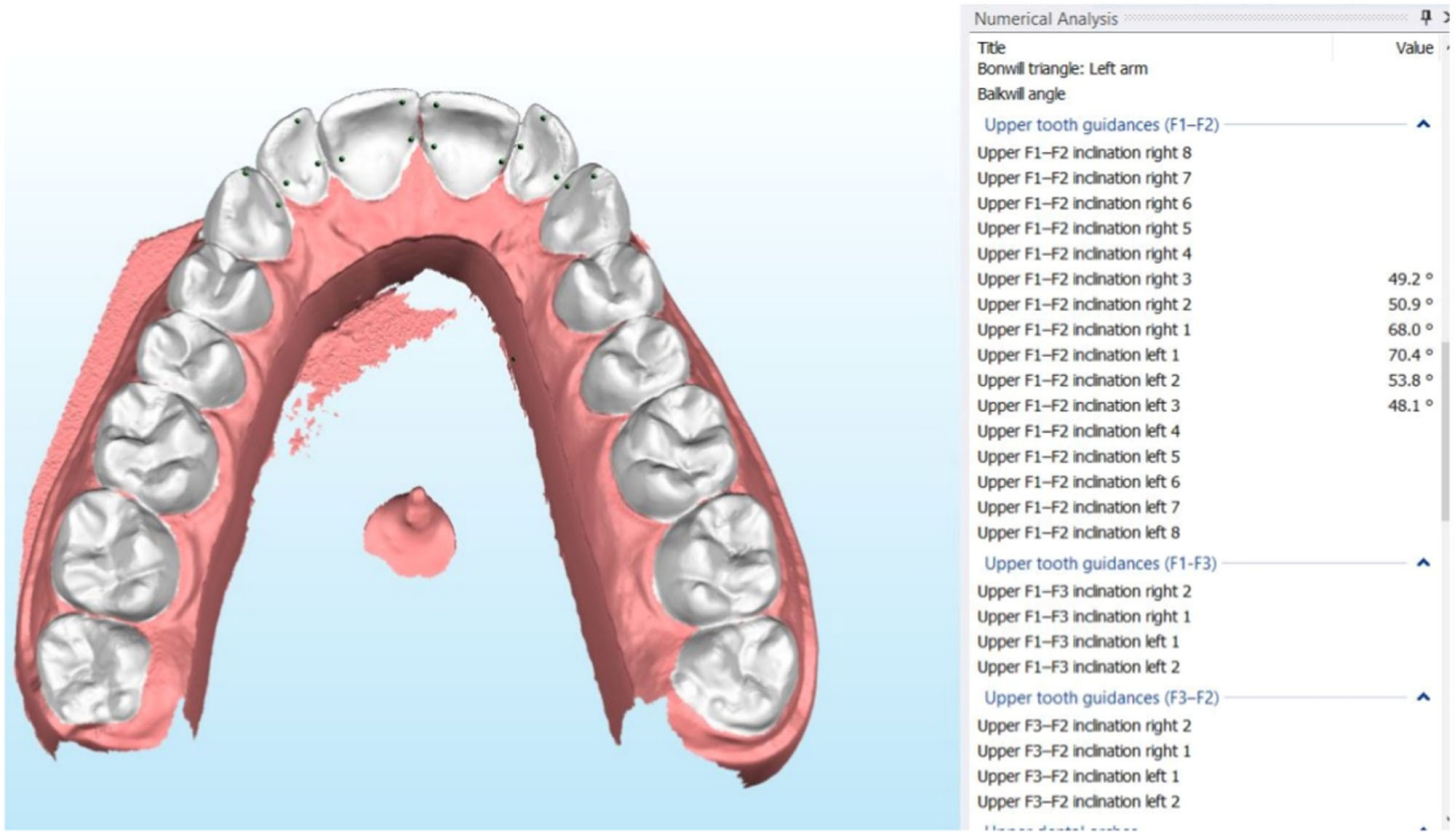
Inclination of anterior teeth. When the corresponding points F1 and F2 are selected, the software displays the numerical data. F1 represents passive centric and F2 represents the point where dynamic lateral and protrusive movement correspondingly ends. The reference plane is the axis–orbital plane.

**Figure 5:**
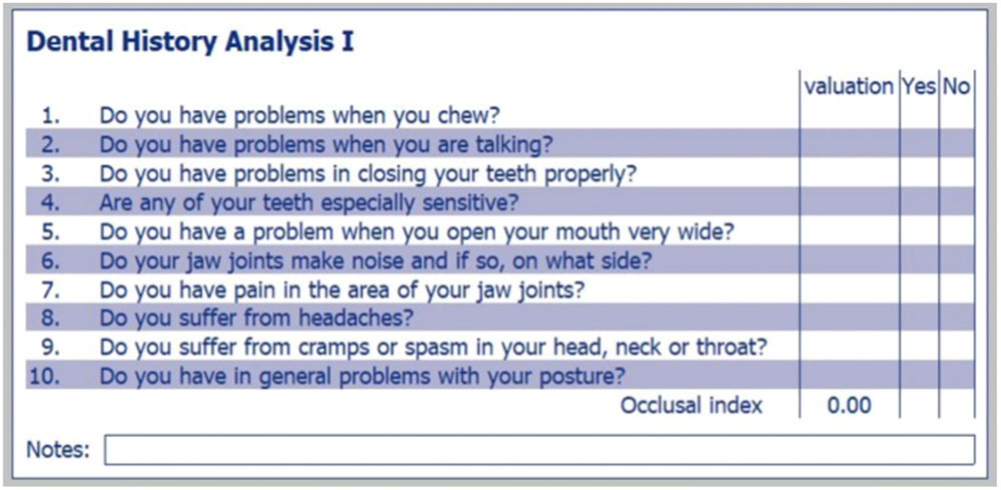
Occlusal index. The patient is asked to answer with a straightforward yes or no, and if a positive answer is derived, the patient is asked to rate the impact from 1 (mild) to 3 (severe). The sum of the positive ratings divided by the number of positive answers provides the index.

For calculating the sagittal condylar inclination (SCI), the average value of three protrusive records was calculated for the 3rd mm of movement for the right and left side. OPI was calculated using the distal cusp of the lower first molars and the incisal edge of the mandibular central incisors. The inclination of anterior teeth was assessed from the contact point (passive centric [Slavicek 2002]) to the point that dynamic lateral and protrusive movement correspondingly ends. If the tooth contact was missing, the inclination was measured from the transition point of the cingulum to the incisal edge. Clinical CD was a part of instrumental functional analysis. From the RP, the patient was instructed to bite in the maximum intercuspal position, and the software calculated the CD. Mechanical CD was calculated digitally, by importing the scanned models in Cadias 3D software and using the Condylar Position Measurement (CPM) option. OI and self‐reported grinding were part of the medical and dental anamnestic. All variables are presented in Table 1 and Figures 1, 2, 3, 4, 5.

**Table 1:** Dependent variables of the study.

Variable	Category	Unit	Assessment
Sagittal condylar inclination	Quantitative	Angle/degrees	Jaw tracking
Inclination of teeth 11, 13, 21, and 23	Quantitative	Angle/degrees	Cadias 3D
Angular difference between SCI and anterior teeth inclination	Quantitative	Angle/degrees	Constructed variable
Occlusal plane inclination	Quantitative	Angle/degrees	Cadias 3D
Clinical condylar displacement in *x*, *y*, *z* axes	Quantitative	Distance/mm	Jaw tracking
Mechanical condylar displacement in *x*, *y*, *z* axes	Quantitative	Distance/mm	Cadias 3D
Grinding awareness	Qualitative‐binary	Yes/no	Self‐report
Occlusal index	Quantitative	0–3	Self‐report
MIDAS score	Quantitative	0–270	Self‐report

*Note:* The category, the measurement unit, and the method by which each variable was assessed are presented.

Abbreviations: MIDAS, migraine disability assessment scale; SCI, sagittal condylar inclination.

### 2.4 Statistical Analysis

Qualitative variables are reported as numbers, while quantitative variables are reported as mean and standard deviation. To determine whether the qualitative variables are independent of the group variable, we used the *χ* ^2^ test. For the quantitative variables, the appropriate normality tests were performed (Kolmogorov–Smirnov or Shapiro–Wilk). To test the effect of the group variable on the values of the quantitative variables, the *t*‐test or the nonparametric Mann–Whitney test was used. Statistical significance was set at 5%. All analyses were conducted using SPSS version 22.0.

## 3 Results

**Figure 6:**
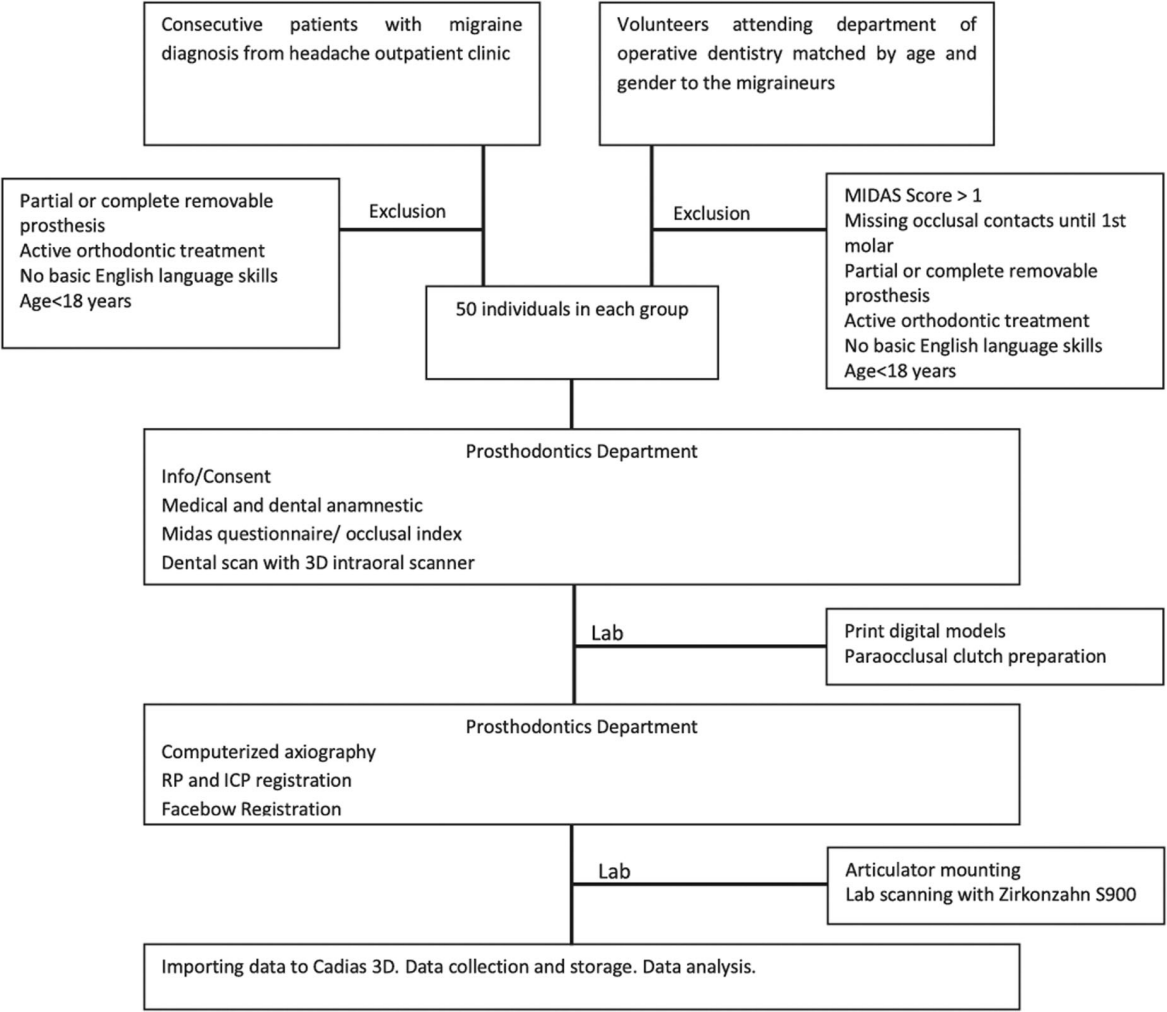
Study flowchart.

The study flow is presented in Figure 6. A total of 50 subjects were recruited in each group. The two groups were matched by age (±5 years) and gender, and each group included 37 females and 13 males. The mean age of the participants was 39 years (40.7 MG, 37.3 CG).

**Figure 7:**
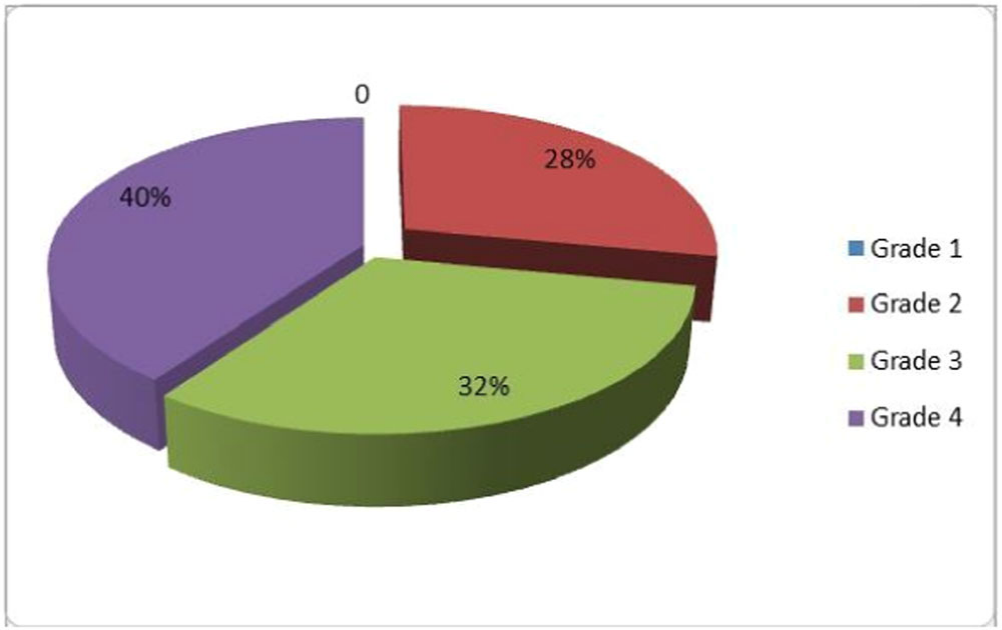
The Migraine Disability Assessment (MIDAS) questionnaire is an instrument that measures the impact that migraine headaches have on patients' life. The information is also helpful for the primary care provider to determine the level of pain and disability caused by migraines and to determine the best treatment for the patient. In this figure, MIDAS categorization of the migraine group is presented.

The Midas score in the MG was 29.48, pain intensity was 6.7 on a 0–10 scale, average headache days per month were 8, and 72% of the patients were categorized in grades 3 and 4 (Figure 7), suggesting medium to severe disability. The OI was 1.92 in the MG, and grinding awareness was reported by almost 80% of the MG.

Regarding CD, a prominent feature of the study, the MG presented statistically significantly higher values of posterior displacement either by clinical or digital‐mechanical means. Furthermore, in terms of direction, retro‐cranial displacement was more prominent in the MG.

The occlusal features examined were all related to the axis–orbital reference plane. Inclination of anterior teeth examined was steeper in the CG, while condylar guidance was steeper in the MG. OPI was also steeper in the MG.

After the statistical analysis, the results revealed that

- Posterior CD, both in clinical and digital‐mechanical measurements, was statistically significantly higher in the migraine group (*p* = 0.012 and *p* = 0.001, respectively) (Table 2).
- Cranial CD in clinical measurements was statistically significantly higher in migraineurs (*p* = 0.046) (Table 2).
- Retral and cranial displacements were statistically significantly more prominent in migraineurs (*p* = 0.025 and *p* = 0.014) (Table 2).
- Angular difference between the steepness of the articular eminence and the contra‐lateral canine guidance was statistically significantly greater in migraineurs (*p* = 0.021 and *p* = 0.017 for right and left laterotrusion) (Table 3).
- OPI was statistically significantly steeper in migraineurs (*p* = 0.024) (Table 4).
- Self‐reported grinding was statistically significantly more often in migraineurs (*p* < 0.001) (Table 4).
- OI was statistically significantly higher in migraineurs (*p* < 0.001) (Table 4).

**Table 2:** Condylar displacement.

		Migraine group		Control group		*p* value for	
	Condylar displacement	*N*	Mean (SD)	*N*	Mean (SD)	Number	Mean
Clinical	Δ*X* anterior (+)	49/94	0.49 (0.67)	64/94	0.29 (0.27)	0.025*	0.251
	Δ*X* posterior (−)	45/94	0.49 (0.67)	30/94	0.29 (0.27)		0.012*
	Δ*Z* caudal (+)	54/94	0.38 (0.38)	66/94	0.38 (0.32)	0.069	0.784
	Δ*Z* cranial (−)	40/94	0.63 (0.73)	28/94	0.31 (0.36)		0.046*
	Δ*Υ*	47/47	0.13 (0.18)	47/47	0.13 (0.11)		0.172
Digital–mechanical	Δ*X* anterior (+)	48/98	1.08 (0.92)	53/100	1.14 (1.06)	0.572	0.973
	Δ*X* posterior (−)	50/98	1.53 (0.95)	47/100	0.90 (0.66)		0.001*
	Δ*Z* caudal (+)	33/98	1.12 (1.01)	51/100	1.21 (0.78)	0.014*	0.314
	Δ*Z* cranial (−)	65/98	1.48 (1.08)	49/100	1.42 (0.96)		0.893
	Δ*Υ*	49/49	0.98 (0.68)	50/50	0.86 (0.74)		0.22

*Note:* The displacement of each condyle from the reference position to the maximum intercuspal position was calculated using two different methods, one clinical during instrumental functional analysis and one mechanical‐digital, using Cadias 3D software. The displacement of each condyle was analyzed in the three axes *x*, *y*, and *z*. Axis *X* represents the antero‐posterior direction, axis *Z* represents the cranio‐caudal direction, and axis *Y* represents the transversal direction. Between migraineurs and controls, a comparison was performed not only for the amount of displacement per direction (column: *p* value for mean) but also for the direction of the displacement (column: *p* value for number).

Abbreviation: SD, standard deviation.

* Significant difference at *p* < 0.05.

**Table 3:** Angular difference in degrees between sagittal condylar inclination and the inclination of the corresponding teeth 11, 13, 21, and 23.

		Absolute values			Actual values		
		Migraine group (SD)	Control group (SD)	*p* value	Migraine group (SD)	Control group (SD)	*p* value
Angular difference	Protrusion right (SCI R minus F1/F2 11)	13.95 (9.46)	12.85 (10.61)	0.368	−8.88 (14.41)	−11.08 (12.49)	0.417
	Laterotrusion right (SCI L minus F1/F2 13)	13.11 (8.33)	9.47 (7.08)	0.021*	9.94 (11.99)	1.95 (11.74)	0.001*
	Protrusion left (SCI L minus F1/F2 21)	11.5 (9.63)	14.73 (10.74)	0.095	−9.33 (11.78)	−13.26 (12.54)	0.11
	Laterotrusion left (SCI R minus F1/F2 23)	12.94 (8.71)	9.44 (8.70)	0.017*	9.58 (12.37)	4.33 (12.15)	0.035*

*Note:* In absolute values, negative signs were not taken into account and the absolute value of the angular difference was used. Sagittal condylar inclination was calculated by means of jaw tracking. The average value of three protrusive records was used for the 3rd mm of movement for the right and left side. Inclination of teeth was calculated using Cadias 3D from the contact point of the antagonist (passive centric‐F1 point) to the point that dynamic lateral and protrusive movement correspondingly ends (F2 point). The reference plane is the axis–orbital plane.

Abbreviations: F1, passive centric; F2, point that dynamic lateral and protrusive movement correspondingly ends; SCI, sagittal condylar inclination; SD, standard deviation.

* Significant difference at *p* < 0.05.

**Table 4:** Difference between groups in the occlusal variables studied.

	Unit	Migraine group (SD)	Control group (SD)	*p* value
SCI R	Degrees	54.49 (7.71)	52.92 (8.55)	0.339
SCI L	Degrees	55.49 (8.29)	50.98 (8.79)	0.01*
SCI R and L	Degrees	54.99 (7.98)	51.95 (8.68)	0.011*
F1–F2 11	Degrees	63.37 (11.06)	64 (9.83)	0.763
F1–F2 13	Degrees	45.55 (9.84)	49.03 (7.76)	0.053
F1–F2 21	Degrees	64.82 (8.82)	64.24 (8.99)	0.745
F1–F2 23	Degrees	44.9 (9.39)	48.59 (8.5)	0.042*
F1–F2 canines	Degrees	45.23 (9.57)	48.81 (8.10)	0.005*
OPI	Degrees	11.16 (4.66)	9.09 (4.37)	0.024*
Grinding awareness	Yes	39/50	12/50	< 0.001*
Occlusal index	0–3	1.92 (0.46)	0.21 (0.66)	< 0.001*

Abbreviations: F1, passive centric; F2, point that dynamic lateral and protrusive movement correspondingly ends; OPI, occlusal plane inclination; SCI, sagittal condylar inclination; SD, standard deviation.

* Significant difference at *p* < 0.05.

## 4 Discussion

In this study, functional occlusal variables were investigated in a group of migraineurs and an age‐ and sex‐matched control group. Statistically significant differences were found for several of them, suggesting that occlusal variables might play a role in migraine disorders. Migraine is a primary headache disorder, so by definition, it is not caused by an organic cause, the dental occlusion included. The comorbidity of migraine and TMDs is well documented in adults (Goncalves et al. 2013; Franco et al. 2010). There also seems to be a connection between bruxism and migraine, with headache patients reporting higher grinding and gnashing behavior (Manfredini et al. 2013; Didier et al. 2014). Favorable results (Goncalves et al. 2013; Didier et al. 2012; Cooper and Kleinberg 2008) have been reported with the use of orthotic appliances in migraine patients, suggesting that implementing neuromuscular gnathological concepts in the treatment of migraine may be beneficial. Under this prism, the investigation of occlusal characteristics both in the dental arch and the condylar level is well justified. In an attempt to explain the pathophysiological connection ‐if any‐ specific occlusal variables could be considered as trigerring or perpetuating factors for migraine headaches.

The migraine group mainly included grade 3 and grade 4 adults, as categorized by MIDAS, suggesting moderate to severe disability for 72% of the sample. This was an expected finding since the pool of patients was from a hospital's outpatient headache neurological clinic. Interestingly, MG presented with the established 3:1 (Mier and Dhadwal 2018) female‐to‐male sex ratio without attempting such a proportion during formation. Additionally, the mean age was 39 years, within the peak decade of migraine disorders.

### 4.1 CD

The most important aspect of this study was CD from RP to ICP. To our knowledge, so far, no other study has investigated the CD in a cohort of migraine patients. Many studies have investigated CD in TMD cohorts and its potential role as an etiologic factor, with contradictory results. In a recent systematic review (Jiménez‐Silva et al. 2017), the authors conclude that due to the high heterogeneity in study designs and the variability of diagnostic methodology, a consistent association between CD and TMDs cannot be determined. Our study presented similar results to the study of He et al. (2010) regarding posterior CD. The mean value of our study was 1.53 mm for migraineurs, while He et al. reported displacements of 1.4 mm for the right side and 1.5 mm for the left side in a group of pretreated orthodontic patients with signs and symptoms of TMDs. According to Cordray (2016), displacements ≥ 1.6 mm in the horizontal plane, ≥ 2.0 mm in the vertical plane, and ≥ 0.5 mm in the transverse plane have been considered clinically significant.

**Figure 8:**
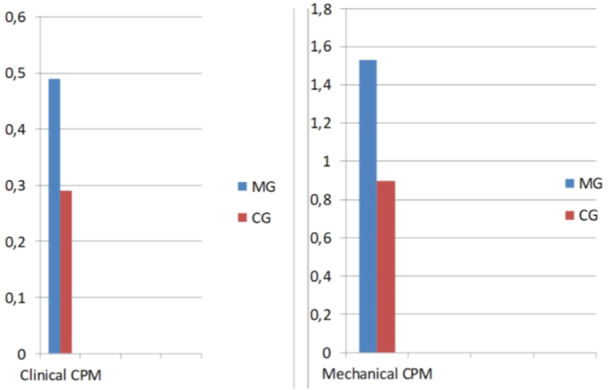
The proportion of retral condylar displacement is very similar, almost 1.7 times higher in migraineurs by both methods, clinical by Jaw tracking and digital‐mechanical by Cadias 3D software.

In every clinical research, the applied methodology and equipment are of paramount importance (Cordray 2017). We calculated CD using two different methods. The first method was part of the instrumental functional analysis, known as clinical CPM. The second method was a laboratory‐bound mechanical technique using an articulator, utilizing RP and ICP records and corresponding equipment extraorally for digitization. Both methods established statistically significant differences between groups in the amount of retral displacement. Cranial displacement presented with statistically significantly higher values in the MG only in the clinical setting, while similar values between groups in the amount of displacement in all other directions were obtained. The fact that mechanical CD resulted in higher values than clinical CPM can be attributed to the clinical assessment performed in a working joint with muscles, ligaments, and the neuromuscular effect. However, the proportional comparison of posterior displacement between groups was almost the same by both methods, 1.7 times (almost 70%) higher in migraineurs (Figure 8).

This consistently higher posterior displacement was an interesting finding, if one considers the sensory innervation of the joint by the auriculo‐temporal and the deep temporal nerves. These are sensory nerves found in the bilaminar zone and the retrodiscal tissues. Nerve endings in the area are Ruffini, Paccini, and free nerve endings responsible for pressure and pain. If noxious stimuli and nociception affect the auriculo‐temporal nerve due to retral CD and/or microtrauma, then various aspects of auriculo–temporal neural interactions could be affected, leading to an array of disorders (Demerjian, Barkhordarian, and Chiappelli 2018; Barkhordarian, Chiappelli, and Demerjian 2018). The pathogenesis of migraine is widely believed to involve peripheral and central activation of the trigeminovascular system. The auriculo–temporal nerve has been identified as one of the peripheral trigger sites for migraine headaches. There are reports in the literature that auriculo–temporal compression is related to migraine headaches (Chim et al. 2012; Sanniec, Borsting, and Amirlak 2016). Retro cranial CD has also been associated with increased activation of the prefrontal cortex and the amygdala, which are connected with emotional and painful neural processing (Greven et al. 2011).

### 4.2 Anterior and Posterior Guidance

Another interesting finding of this study was that the angular difference between the condylar inclination of the mediotrusive path(s) and the contralateral canine inclination was statistically significantly greater in migraine patients. In general, migraine patients presented with steeper condylar inclination and smoother canine guidance, contributing to an even higher angular difference. In the literature, values of SCI vary from 40° to 58.6° (Zoghby, Ré, and Perez 2009) depending on the method and the reference plane used. The same principle applies for calculating the inclination of anterior guidance; methods and reference plane affect the numerical data derived. Celebic et al. (2007) report 57.5° incisal guidance and 50° condylar guidance, but relative to the Frankfort horizontal plane, which explains the lower values compared to our study. Isberg and Westesson (1998) measured in MRIs the inclination of the condylar path and the steepness of the articular eminence in relation to the horizontal plane. They reported that the steeper the eminence, the more prominent the posterior disc rotation. Inclination of the condylar path was 61.1 degrees. Incisal and canine guidance may be expressed in degrees relative to a reference plane. The inclination can be assessed from the contact point (passive centric) to the point that dynamic lateral and protrusive movement correspondingly ends (Slavicek 2002). If the tooth contact is missing, the inclination can be measured from the transition point of the cingulum to the incisal edges (Donegan and Knap 1995). Donegan and Knap (1995) reported no statistical difference in the inclination of the functioning surfaces of maxillary anterior teeth. In their study, central incisors were inclined by 46° and canines by 44° relative to the occlusal plane, so one would have to add the OPI relative to the axis–orbital plane to make it comparable to our results.

Anterior and posterior guidance are decisive factors of mandibular kinematics, and are interdependent. Development of the joint follows function. In a newborn, the articular eminence is flat. It is developed during growth by the functional influence of a dynamic occlusion and responds to functional influences throughout life (Slavicek 2002). The functional coordination of the TMJ and occlusion is important in maintaining a healthy masticatory system (Li et al. 2021). Different anterior and posterior guidance ratios have been found to affect the activity of anterior and posterior temporal muscles (Celebic et al. 2007). Too steep anterior guidance causing hinge‐opening rotation of the mandible during protrusion creates a conflict between the muscles and the incisal guidance. It is related to abnormal movements in the joint (Thompson 1986). Steep anterior guidance may also be related to avoidance patterns. Some signs of TMD occur more often in patients with increased vertical overlap anteriorly and minimal horizontal overlap (Tinastepe and Oral 2015). On the other hand, if anterior guidance is flatter than the posterior path, the probability of functional interferences increases (Bell and Harris 1983). Belser and Hannam (1985) investigated the influence of laterotrusive and mediotrusive occlusal guidance on EMG activity of masticatory muscles and related jaw movement by inserting artificial overlays on canines and molars. They concluded that mediotrusive interferences dramatically altered the distribution of muscle activity during parafunctional clenching. This redistribution may affect the nature of reaction forces at the temporomandibular joints.

### 4.3 OPI

Another finding of this study was the statistically significant difference in the inclination of the occlusal plane, which seemed to be flatter in the CG. There was about a 3° angular difference, with inclination in the CG being around 9° and almost 12° in the migraineurs. Values in the literature vary from 6° to 10° depending on how the occlusal plane is defined and the reference plane used. The configuration of the occlusal plane is crucial since it forms the basis by which occlusal surfaces of teeth can be related to each other and other head structures. The occlusal plane's level and inclination result from the neuromuscular growth and developmental forces acting on the dentition. Inclination of the occlusal plane seems to be essential for head posture (Chan 2007) and mastication (Sato et al. 2007; Ogawa, Koyano, and Umemoto 1998). The spatial relationship of the occlusal plane, compensating curves, anterior guidance, and SCI can indicate susceptibility of disturbances to functional occlusion (Bumann and Lotzmann 2002). Slavicek has suggested a formula for deciding on the OPI, according to which, the angle of disocclussion and the SCI are taken into account (Slavicek 2002). The higher the angle of disocclussion, the lower the probability for interferences, the flatter the occlusal plane for a given SCI and cusp inclination. In simple words, if the occlusal plane is too high in the back, the posterior teeth may interfere with anterior teeth guidance.

### 4.4 Subjective Findings

In the medical and dental anamnestic, both self‐reported grinding and OI were found to be statistically significantly higher in the migraine group. The OI is based on ten questions evaluating the patient's subjective symptoms concerning the stomatognathic system (Gsellmann et al. 1998). The patient is asked to answer with a straightforward and spontaneous yes or no, and if a positive answer is derived, the patient is asked to rate the impact from 1 to 3, with 1 representing mild, 2 representing moderate, and 3 representing severe impact on quality of life. The sum of the positive ratings divided by the number of positive answers provides the OI; thus, the index ranges from 0 to 3. The number of positive questions may also be interpreted, suggesting if the patients' problem is focused or multilayered. The higher the OI, the more susceptible the patient is to functional problems. Higher OI in the MG is in accordance with other findings of this study. It suggests the need to consider the function of the stomatognathic system in treating migraine patients.

The association between bruxism and migraine has been a subject of interest in recent years, with numerous papers indicating a positive association (Peşkersoy et al. 2016; Didier et al. 2014; Zaproudina et al. 2018a; Muayqil et al. 2018; De Luca Canto et al. 2014; Costa et al. 2016). In a systematic review by De Luca Canto et al. (2014), adults with sleep bruxism (SB) appear to be more likely to have headaches. However, only two papers were finally selected since most preselected studies did not meet the minimum criteria for SB and or headache diagnoses. Fernandes (Manfredini et al. 2013) reported a statistically significant association between SB and chronic migraine. Didier et al. (2014) also reported a statistically significant correlation between parafunction and chronic migraine. Kato et al. (2016) reported extensive grinding in migraineurs, especially in the molar region, by utilizing bruxcheckers. Our findings are comparable to those reported by Didier, who also carried out a self‐reported evaluation of SB.

### 4.5 Overall Judgment

The findings of this study suggest that the MG presented occlusal characteristics that favor the presence of occlusal interferences. If occlusal interferences do exist, it can be challenging to identify since the system may develop avoidance patterns. Avoidance patterns may correspond to different muscle engrams and condylar paths, which in turn may trigger a series of events involving the trigeminovascular complex. The relationship between functional occlusal interferences and TMDs is a complex one. In a population‐based approach, they may not seem to be vital. However, in a patient‐based approach, their importance may increase as the tolerance capacity of the stomatognathic system differs inter‐personally or intra‐personally from time to time (Bell et al. 2002). In our study, the occlusal features of the MG, for example, the steeper occlusal plane and the flatter canine guidance, raise the probability of functional interferences compared to the CG in a pure biomechanical sense. Furthermore, such findings may explain the beneficial effect of splint usage in migraine patients that repeatedly has been reported (Goncalves et al. 2013; Didier et al. 2012; Cooper and Kleinberg 2008).

It is beyond the scope of this research paper to review the importance of occlusion and its potential effect on the stomatognathic system. The connection is still controversial. An excellent way to illustrate the controversy is by just comparing the titles of two recent reviews by two renowned experts in the field: *Temporomandibular Disorders—Occlusion Matters* (de Kanter, Battistuzzi, and Truin 2018), published in 2018, and *Temporomandibular disorders and dental occlusion. A systematic review of association studies: end of an era* (Manfredini, Lombardo, and Siciliani 2017), published in 2017. Even more surprising is the fact that, in an attempt to explore and compare the literature used in these reviews, only one paper is cited in common. In other words, among 189 papers that were cited in total—Manfredini et al. cite 83 papers and De Kanter et al. cite 106 papers—only one was used by both researchers. The mutually cited reference is by Ash (2001) *Paradigmatic shifts in occlusion and temporomandibular disorders*, published in 2001, which also happens to be a review and not a primary research paper.

The present study has limitations. First, this was not a blinded study. The operator was aware of the group of the individual examined. However, the fact remains that objective measurements were performed and all the procedures were carefully conducted by the same experienced clinician for both groups, making this limitation tolerable. Even though digital equipment offers the possibility for precise measurements and analysis, an instrumental functional analysis at the condylar level is definitely operator dependent. The main clinical researcher of this study was trained in the field of instrumental functional analysis and is familiar with the equipment used spanning a long time. Finally, an attempt to repeat measurements after a short period to verify the consistency of findings would have been useful; however, using such equipment is labor‐intensive, making it challenging to obtain consent for a second analysis in a short period. This also justifies why a second examiner did not perform the same operation on the same patient twice.

The present study also has strengths. It was conducted in close adherence to the STROBE statement (Strobe-statement.org, n.d.) for cross‐sectional studies. Sample selection was carefully designed, considering the power of the study. All patients in the migraine group had been diagnosed by a neurologist according to the IHS criteria. We also included an age‐ and gender‐matched control group. Measurements from 3D intraoral scanner, a 3D lab scanner, accurate hinge axis determination, and Cadias 3D software offered precision and objective values. The same operator performed all clinical and technical procedures for standardization and to eliminate inter‐operator error.

## 5 Conclusions

The null hypothesis of this study was rejected. Statistically significant differences were found for several functional occlusal variables between individuals with migraines and those without migraines. In the context of this study, the function of the stomatognathic system, as affected by occlusal and articular structures, should not be overlooked in an interdisciplinary approach to migraine management.

## Author Contributions

Zokaris Nikolaos conceived the study, designed the protocol, prepared and collected data, wrote the manuscript, and contributed to the interpretation of the results. Greven Marcus contributed to study design, interpretation of the results, and critical revision of the manuscript. Naoumis Dimitrios examined the migraine patients as a consultant in 251 Hellenic Air Force and VA Hospital. Tzakis Michail and Mitsikostas Dimos Dimitrios contributed to study design. Psarras Vasileios contributed to study design, interpretation of the results, and critical revision of the manuscript for intellectual content and approval of the final version.

## Conflicts of Interest

The authors declare no conflicts of interest.

## Data Availability

The data that support the findings of this study are available from the corresponding author, N.Z., upon reasonable request.
